# Viral Communities Associated with Human Pericardial Fluids in Idiopathic Pericarditis

**DOI:** 10.1371/journal.pone.0093367

**Published:** 2014-04-01

**Authors:** Laura Fancello, Sonia Monteil, Nikolay Popgeorgiev, Romain Rivet, Frédérique Gouriet, Pierre-Edouard Fournier, Didier Raoult, Christelle Desnues

**Affiliations:** Unité de recherche sur les maladies infectieuses et tropicales émergentes, URMITE CNRS-IRD UMR 7278, Aix-Marseille Université, Marseille, France; Faculty of Biochemistry Biophysics and Biotechnology, Jagiellonian University, Poland

## Abstract

Pericarditis is a common human disease defined by inflammation of the pericardium. Currently, 40% to 85% of pericarditis cases have no identified etiology. Most of these cases are thought to be caused by an infection of undetected, unsuspected or unknown viruses. In this work, we used a culture- and sequence-independent approach to investigate the viral DNA communities present in human pericardial fluids. Seven viral metagenomes were generated from the pericardial fluid of patients affected by pericarditis of unknown etiology and one metagenome was generated from the pericardial fluid of a sudden infant death case. As a positive control we generated one metagenome from the pericardial fluid of a patient affected by pericarditis caused by herpesvirus type 3. Furthermore, we used as negative controls a total of 6 pericardial fluids from 6 different individuals affected by pericarditis of non-infectious origin: 5 of them were sequenced as a unique pool and the remaining one was sequenced separately. The results showed a significant presence of torque teno viruses especially in one patient, while herpesviruses and papillomaviruses were present in the positive control. Co-infections by different genotypes of the same viral type (torque teno viruses) or different viruses (herpesviruses and papillomaviruses) were observed. Sequences related to bacteriophages infecting Staphylococcus, Enterobacteria, Streptococcus, Burkholderia and Pseudomonas were also detected in three patients. This study detected torque teno viruses and papillomaviruses, for the first time, in human pericardial fluids.

## Introduction

Pericarditis is defined by inflammation of the pericardium, the sac-like membrane surrounding the heart. Pericarditis can be of non-infectious or infectious origin. Non-infectious pericarditis, which represents approximately 1/3 of cases, can be due to autoimmune or neoplastic diseases, metabolic disorders or traumas [1]. The infectious forms of pericarditis are mainly of viral or bacterial origin. Mycobacterium tuberculosis is the most frequently diagnosed bacterial agent and usually occurs in developing countries or immune-compromised hosts [1], [2]. In Western countries, the large majority of acute pericarditis cases are viral, and echovirus, coxsackievirus, influenza, Epstein-Barr virus, cytomegalovirus, adenovirus, varicella, rubella, mumps, hepatitis B, hepatitis C, human immunodeficiency virus, parvovirus B19 and human herpesvirus 6 are the principal infectious agents [1]–[3].

Determination of pericarditis etiology is difficult, and a large number of cases remain unexplained. Indeed, idiopathic pericarditis represents between 40% and 85% of pericarditis cases, and most of them are suspected to be of viral origin [2], [4]. Because serological tests are only suggestive and not diagnostic, the diagnosis of viral pericarditis requires invasive methods that evaluate pericardial effusion or tissue [3]. For example, in a study evaluating the etiology of 204 pericarditis cases, Levy et al. showed that no statistically significant difference between patients and controls could be observed in serological tests targeting adenovirus, influenza virus or cytomegalovirus, thus showing that the specificity of some of these tests is very low [5]. Moreover, classical diagnostic techniques do not allow the detection of unknown viruses or already known viruses not suspected of involvement in the disease. Thus, an “a priori-free” approach needs to be applied to clarify the etiology of idiopathic pericarditis cases. Recently, the advent of next generation sequencing technologies has allowed the development of a metagenomic approach for a more complete and unbiased view of viral communities associated with a sample. This approach has already been successfully applied to human clinical studies and has led to the discovery of new potential pathogenic viruses as well as the identification of unsuspected viruses in some idiopathic diseases [6]–[13]. In the work presented here, we applied a metagenomic approach to investigate the DNA viral community colonizing human pericardial fluids and identify unsuspected or new, divergent viruses likely to be responsible for unexplained cases of pericarditis.

To this end, we generated viral DNA metagenomes of pericardial fluid samples collected from 7 patients affected by idiopathic pericarditis and from 1 patient as part of sudden infant death protocol. We compared these metagenomes to 2 metagenomes generated from individuals affected by pericarditis of non-infectious origin, used as negative controls.

## Materials and Methods

### Samples collection

A total of 15 pericardial fluid samples were used in this study. All samples were collected between 2007 and 2010 in 5 French hospitals (the Hospital of Niort and Timone, Nord, Conception and Clairval hospitals in Marseille). Samples collection was carried out in an operating room, under strictly sterile conditions. The pericardial punction was achieved without piercing any other organ than the skin. Except for patient P4, samples P1 to P8 were collected from patients affected by pericarditis of unknown etiology (i.e., classical diagnostic tests performed at the hospital were all negative). Patient P4 was included as part of a sudden infant death protocol, although the culture showed a polymicrobial infection (Escherichia coli and Pseudomonas species). One sample (the positive control) came from a patient affected by a pericarditis caused by human herpesvirus 3. Six samples (forming the negative controls N1 and N2) came from different individuals affected by pericarditis of non-infectious origin (i.e., traumatic or post-surgery). These samples were treated separately. Then the amplified viral DNAs generated for 5 of them were pooled together prior to sequencing (pool negative controls N1), whereas that of the remaining sample was sequenced alone (negative control N2). Volumes of available pericardial fluids varied between approximately 130 μl and 2 ml. Samples were conserved at −80°C until processing. All samples are listed in Table 1 along with the age and gender of patients and hospital diagnosis.

**Table 1:** Samples, diagnosis and metagenomic results.

Sample	Age^a^	Sex	Metavir identifier^b^	Diagnosis	Pathological agent (hospital diagnostic tests)	Most abundant viral type in viral metagenomes^c^
P1	34	M	LPC_P1	Idiopathic pericarditis	-	*Anelloviridae* (57.04%) *Retroviridae* (35.78%) Bacteriophages (4.74%)
P2	73	F	LPC_P2	Idiopathic pericarditis	-	*Anelloviridae* (68.76%) *Retroviridae* (29.68%)
P3	81	M	LPC_P3	Idiopathic pericarditis	-	Bacteriophages (33.69%) *Anelloviridae* (26.05%)
P4	6 months	M	LPC_P4	Sudden infant death	Polymicrobial infection	Bacteriophages (49.32%) *Retroviridae* (31.07%)
P5	66	M	LPC_P5	Idiopathic pericarditis	-	*Retroviridae* (90.51%)
P6	43	F	LPC_P6	Idiopathic pericarditis	-	*Anelloviridae* (60.23%) Retroviridae (28.58%)
P7	64	M	LPC_P7	Idiopathic pericarditis	-	*Anelloviridae* (98.59%)
P8	88	M	LPC_P8	Idiopathic pericarditis	-	*Retroviridae* (90.9%)
						
Positive control	19	F	LPC_PosContr	Viral pericarditis	Human herpesvirus 3	*Herpesviridae* (66.25%) *Papillomaviridae* (30.28%)
						
Pool negative controls N1	6	M	LPC_PoolNegContrN1	Pericarditis of non infectious origin d	-	*Retroviridae* (90.85%) d
Pool negative controls N1	87	F	LPC_PoolNegContrN1	Pericarditis of non infectious origin d	-	*Retroviridae* (90.85%) d
Pool negative controls N1	49	M	LPC_PoolNegContrN1	Pericarditis of non infectious origin d	-	Retroviridae (90.85%) d
Pool negative controls N1	7 months	F	LPC_PoolNegContrN1	Pericarditis of non infectious origin d	-	*Retroviridae* (90.85%) d
Pool negative controls N1	3	M	LPC_PoolNegContrN1	Pericarditis of non infectious origin d	-	*Retroviridae* (90.85%) d
Negative control N2	7	F	LPC_NegContrN2	Pericarditis of non infectious origin	-	*Retroviridae* (53.08%) *Anelloviridae* (41.5%)

a In years, if not indicated otherwise.

b Identifier on the Metavir server (http://metavir-meb.univ-bpclermont.fr) for preprocessed viral metagenomes.

c The most abundant viral type in the associated viral metagenome according to the GAAS analysis.

d Samples were pooled together and one unique viral metagenome was generated.

### Ethics Statement

All samples used in this study were collected from human subjects using a protocol approved by the local ethics committee IFR48 (Marseille, France). Written informed consent was obtained from the parents or legal guardians of all subjects.

### Viral isolation and sequencing

Each sample was centrifuged at low speed to eliminate proteins and cellular debris. The resulting supernatant was collected and filtered through 0.45-μm filter pore. Virus-like particles were concentrated by ultracentrifugation at 55,000 g for 60 min. The resulting pellet was resuspended in a phosphate buffered saline solution (PBS) previously filtered at 0.02 μm. Purified VLPs were treated with DNAse and RNAse to remove any residual host and bacterial DNA as previously described [14].

Standard PCR reactions targeting human 18S rDNa were performed to verify the absence of host DNA contamination. Viral DNA was then extracted using the High Pure Viral Nucleic Acid Kit (Roche Applied Science, Inc, Branford, CT) following the manufacturer's recommendations. Extracted DNA was amplified using the commercial Illustra™ GenomiPhi V2 DNA Amplification Kit (GE Healthcare Life Sciences, Freiburg, Germany) to generate sufficient material for shotgun 454 pyrosequencing library preparation. Amplified DNA was purified on silica columns (Qiagen Inc, Valencia, CA) to remove the enzyme, dNTPs and primers, and subsequently sequenced on a 454 Life Sciences Genome Sequencer FLX instrument using titanium chemistry (Roche Applied Science, Inc, Branford, CT).

All reagents, except for those from kits, were filtered at 0.02 μm before use to prevent any potential contamination.

### Reads pre-processing and annotation

The generated sequences were screened to remove the exact and nearly identical duplicates using the CD HIT 454 program [15], available under the CAMERA 2.0 web portal [16]. All screened viromes are publicly available on the Metavir web-server (Table 1) [17]. A BLASTN search against the non-redundant NCBI database (E<1e^−05^) was performed. Reads having no significant hits were classified as “unknown reads”, whereas those with significant similarity to sequences stored in the NCBI database were classified as “known reads”.

### Estimation of viral genotype abundances

The GAAS (Genome relative Abundance and Average Size) program [18] was used to estimate the relative abundance of each viral genotype present in the metagenomes. Briefly, GAAS performs a BLASTX search against the Viral Refseq database and normalizes the number of reads matching one viral genotype to the genome length of that viral genotype. The BLASTX search was performed with an E-value of 1e^−05^.

### Mapping

Reads were mapped onto reference genomes using the CLC Genomics Workbench version 4.9 (www.clcbio.com) with a minimal length fraction of 0.5 and a minimal similarity of 0.8 as mapping parameters.

### Assembly and contig analysis

Read assemblies were performed for each sample using the GS *De Novo* Assembler (Roche Applied Science, Inc, Branford, CT), an application especially suited for the analysis of the 454 Life Sciences Genome Sequencer FLX-generated data. We chose a minimum overlap length of 35 bp and a minimum overlap identity of 98%. Only contigs longer than 400 bp were kept for further analyses. We classified as “large contigs” those spanning more than 1,500 bp. Contigs were classified as known if they had a significant hit in a BLASTN search against the non-redundant NCBI database (E-value<1e^−05^), otherwise they were classified as unknown. Contigs were then annotated by a BLASTX search against the non-redundant NCBI database (E-value<1e^−05^).

### PCR for specific sequences

To confirm the presence of sequences found *in silico*, standard PCRs targeting specific viral genotypes recovered by the GAAS analysis were performed. All primers used are listed in Table S1.

#### Torque teno viruses (TTV)

Genomiphi-amplified DNA extracted from sample P7 was tested for torque teno virus presence by nested PCR using the primers previously described by Peng et al., which detect all genotypes and genetic groups currently identified for torque teno viruses [19]. These primers target the UTR universally conserved region of TTV genomes. Nested PCR was carried out with Phusion High-fidelity DNA Polymerase (Finnzymes Oy, Thermo Fisher Scientific) using 1 μl of template DNA. Three specific primer pairs were designed using Primer3 [20] to target the 3 longer TTV-like contigs assembled from sample P7. Standard PCRs with the designed primers were performed using Phusion High-fidelity DNA Polymerase (Finnzymes Oy, Thermo Fisher Scientific) on the remaining amplified viral DNA for sample P7; specific PCR products were purified and sequenced by Sanger technology for verification.

#### Enterobacteria, Staphylococcus, Pseudomonas, Burkholderia and Streptococcus bacteriophages

Primers from literature were used when available [21], [22]. Otherwise, specific primers for the bacteriophages detected in silico were designed by Primer-BLAST (http://www.ncbi.nlm.nih.gov/tools/primer-blast/), PhiSigns [23] or Primer3, followed by BLAST verification of primers' specificity [20]. PCRs were performed on the Genomiphi-amplified DNA from each sample.

## Results

**Figure 1:**
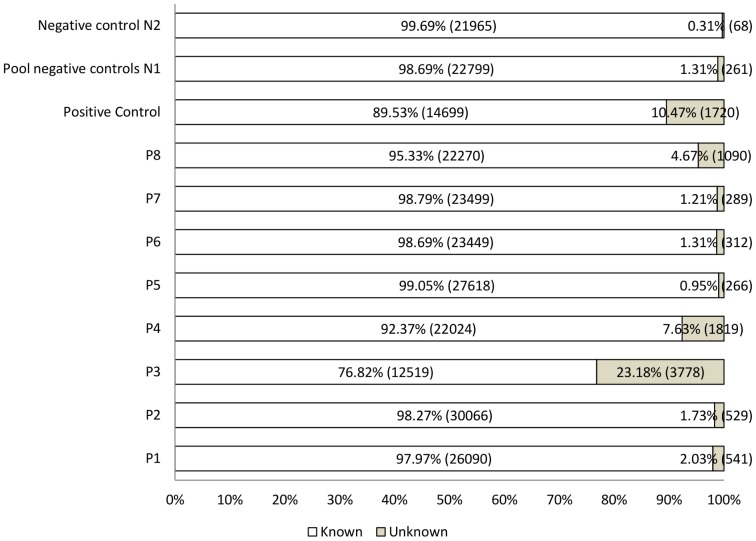
Proportion of known and unknown reads. The chart shows the proportion of metagenomic reads classified as “known” or “unknown,” according to a BLASTN search against the non-redundant NCBI database (E-value<1e^−05^). For each sample, known reads (in white) and unknown reads (in grey) are reported as both a percentage and absolute number (in parentheses).

**Figure 2:**
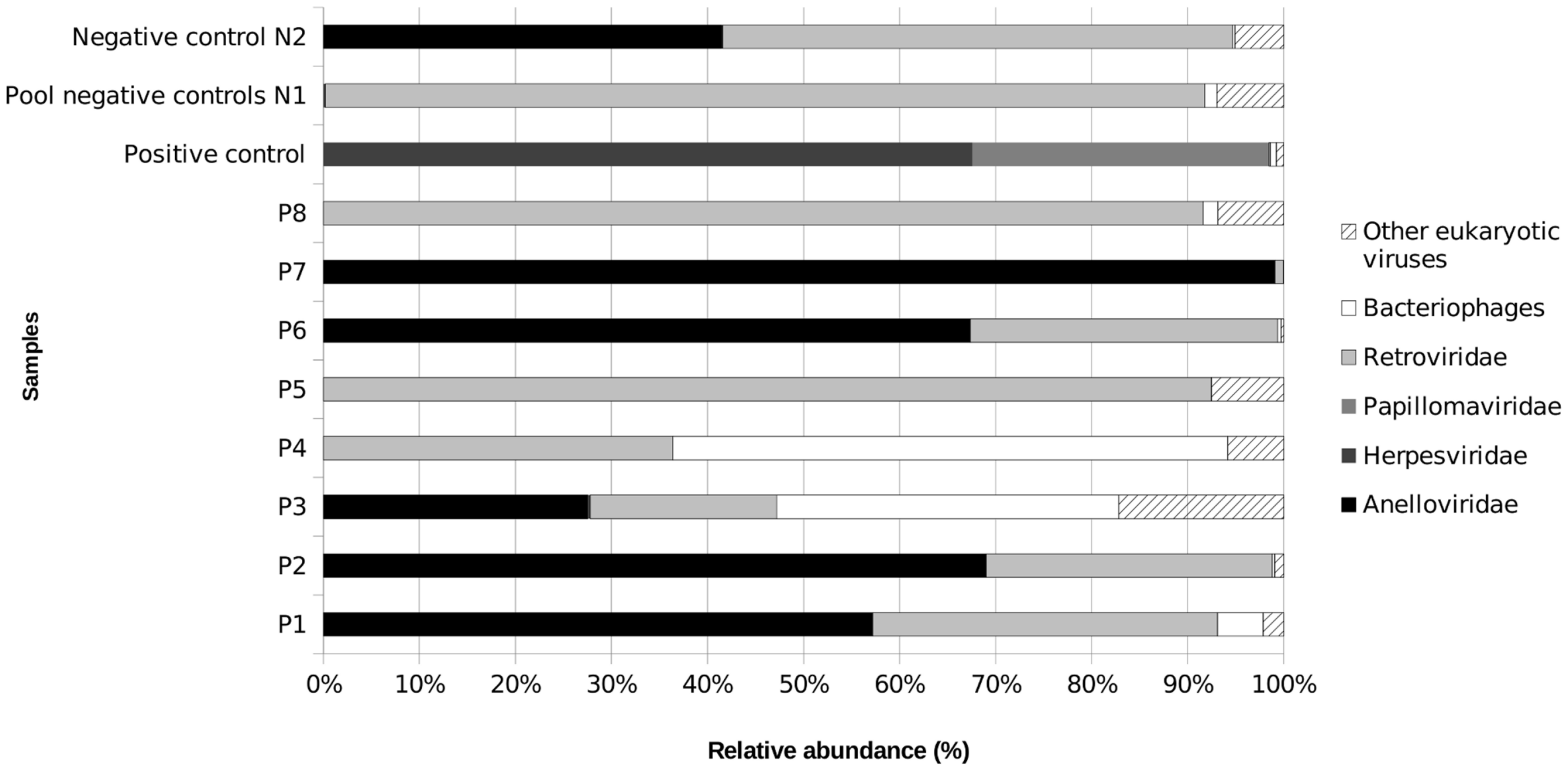
Viral relative abundance in each virome. The estimated relative abundance for each viral group detected in a sample is shown. The data were generated by GAAS analysis. Eukaryotic viral genotypes are grouped together at the family level. All bacteriophages are grouped together in the “Bacteriophages” category.

In this study, 11 viral metagenomes were generated from human pericardial fluid samples. Viral-like particles were purified and treated with DNAse and RNAse to minimize contamination by host and bacterial DNA as previously described [14]. Host contamination was verified by PCR targeting human 18S rDNA. Reactions performed on patient samples, the positive control and five negative controls were negative. Only the reaction performed on one negative control from the pool of negative control N1 presented traces of human contamination. Viral DNA was extracted, amplified in a sequence-independent manner and sequenced using the 454/Roche pyrosequencing technology. A total of 336,461 reads were generated. After removal of artificial duplicates, a total of 257,671 reads were left with an average length between 246 and 361 bp, depending on the sample (Table S2). According to a BLASTN search (E-value<1e-05) against the non-redundant NCBI database, between 0.31% and 23.18% of the reads had no significant similarity to known sequences and were thus classified as “unknown” (Fig. 1). GAAS [18] was used to more accurately estimate the relative abundance of each viral genotype (Fig. 2). Because longer genomes have higher probabilities of being sequenced in the shotgun approach, they are overrepresented in terms of number of sequences in the metagenome. To avoid this bias, GAAS normalizes the number of sequences associated with a viral genotype to its genome length. Relative abundances estimated by GAAS were synthesized and presented at the viral family level except for all bacteriophage-related sequences, which were grouped together. Reads assembly was performed on each metagenome and a total of 2,000 contigs were obtained, including 240 large contigs (longer than 1,500 bp). Depending on the sample, up to 76.66% of the reads were assembled into contigs, which were annotated on the basis of their best hit in a BLASTX search against the non-redundant NCBI database (E-value<1e-05). The total number of contigs and large contigs generated for each sample as well as the percentage of assembled reads is reported in Table S3.

### Human herpesvirus 3 and papillomaviruses co-infection

**Figure 3:**
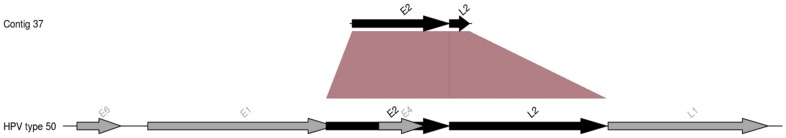
Reconstructed contig matching a human papillomavirus. Contig 37 assembled from the positive control is shown and compared to the genome organization of human papillomavirus type 50. The arrows represent open reading frames (ORFs). Homologous ORFs between the contig and human papillomavirus type 50 are shown in black.

As expected, the positive control showed high abundance of Herpesviridae (6,674 reads) and, in particular, reads matching human herpesvirus 3 (HHV3). Indeed, 5,990 reads related to HHV3 were recovered (BLASTX against the Viral Refseq Genomes Database, E-value<1e-05). After mapping of the reads onto the reference HHV3 genome, 98% (122,502 bp) of the genome was reconstructed (Figure S1) with an average coverage depth of 18.93 reads. In addition to HHV3, 173 reads matching viruses from the Papillomaviridae family were recovered and 11 contigs could be generated after de novo assembly (Table 2). Contig annotation retrieved 4 different types of human papillomaviruses (HPVs): HPV isolate 915 F 06 002 KN1, HPV type 50, HPV type 80, and HPV type 12 (Table 2). Major and minor capsid proteins as well as replication and regulatory proteins were encoded by the contigs (BLASTX against the non-redundant NCBI database, E-value<1e-05). One contig (contig 37, 1196 bp long) was found to harbor two different ORFs matching the E2 and L2 genes of HPV type 50 (BLASTX against the non-redundant NCBI database, E-value<1e-05). Both ORFs were incomplete, but their position on the contig was consistent with that of the E2 and L2 genes on the HPV type 50 genome (Figure 3). More than 50% of each of the four HPV genomes could be reconstructed by mapping (Figure S2 and Table S4), and the subsets of reads that mapped onto each reference genome were mutually exclusive, supporting the presence of multiple different genotypes of papillomaviruses. All recovered HPV genotypes were HPVs first isolated from human skin under healthy (HPV type 80, HPV isolate 915 F 06 002 KN1) or disease (HPV type 50, HPV type 12) conditions [24]–[27].

**Table 2:** *De novo* contigs matching *Papillomaviridae* from the viral metagenome associated with the positive control.

Contig ID	Contig length (bp)	Number of reads^a^	Best Blast hit^b^	E-value	Percent identity	Alignment length (aa)
00017	650	8	major capsid protein L1 [Human papillomavirus type 50]	4e-106	98.39	186
00019	677	6	major capsid protein L1 [Human papillomavirus type 50]	5e-129	98.22	225
00027	487	7	replication protein E1 [Human papillomavirus type 50]	4e-65	87.12	132
00033	645	6	E1 protein [Human papillomavirus]	9e-111	96.91	162
00035	428	2	L2 [Human papillomavirus type 80]	3e-68	98.35	121
00037	1196	19	regulatory protein E2 [Human papillomavirus type 50]	3e-159	96.59	323
00049	787	5	Replication protein E1 [Human papillomavirus type 12]	1e-122	100.00	121
00062	1210	15	replication protein E1 [Human papillomavirus type 50]	1e-144	97.71	175
00080	665	4	E2 protein [Human papillomavirus]	4e-56	77.69	121
00082	771	10	minor capsid protein L2 [Human papillomavirus type 50]	7e-128	96.85	254
00089	461	4	L2 protein [Human papillomavirus]	5e-68	90.26	154

a Number of reads assembled in the contig.

b Best BLAST hit according to a BLASTX search against the non-redundant NCBI database, E<1e^−05^.

### Human endogenous retroviruses

In contrast to the positive control, the negative controls N1 and N2 contained only one read each that matched a herpesvirus and none related to papillomaviruses. The viral DNA community of the negative controls N1 and N2 was dominated by sequences from the Retroviridae family (90.85% and 53.08% respectively) (Figure 2) and no contig could be reconstructed for any eukaryotic virus or bacteriophage. A similar profile was observed for the viral communities of patients P5 and P8. Indeed, viromes associated with these samples were dominated by Retroviridae (90.51% and 90.9% for P5 and P8, respectively) (Figure 2) and no viral contigs could be reconstructed. Analysis of the Retroviridae-related sequences (reads and contigs) identified them as human endogenous retroviruses.

### Variable *Anelloviridae* genotypes were detected in 5 patients

**Figure 4:**
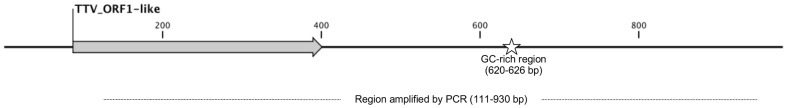
Contig 129 from sample P7. The contig was assembled *de novo* from the viral metagenome associated with sample P7 and matches a torque teno virus. A grey arrow indicates an identified ORF homologous to the ORF1 of torque teno virus. A star indicates a GC-rich region (85.7%). The dotted line under the contig shows the region that was amplified by PCR and sequenced.

According to GAAS analysis, six metagenomes (P1, P2, P3, P6, P7 and negative control N2) contained *Anelloviridae* (57.04%, 68.76%, 26.05%, 60.23%, 98.59% and 41.5%, respectively) (Figure 2) and, in particular, torque teno viruses (TTVs) (49.6%, 66.97%, 26.05%, 60.23%, 98.59% and 38.14%, respectively). In total for *Anelloviridae* (torque teno viruses and others), we recovered 20 reads for P1, 108 for P2, 3 for P3, 56 for P6, 1628 for P7 and 17 for negative control N2 (BLASTX search against the non-redundant NCBI database, E-value<1e^−05^, Table S5). Among the recovered reads, there were reads matching unclassified *Anelloviridae* as well as reads matching Alfatorqueviruses, Betatorqueviruses and Gammatorqueviruses. More than 70% of several TTV genomes could be reconstructed by mapping reads from samples P2, P6 and P7. These genomes corresponded to the TTV clone Saf-09 in sample P2 (Figure S3) and the TTV strain SIA109 and TTV 24 clone Saa-01 in sample P6 (Figure S4). In addition, we were able to fully reconstruct the genome of three TTV isolates (torque teno virus TTV-HD14 h, torque teno virus isolate TTVyon-LC011 and torque teno virus isolate JT41F), the genome of TTV 29 and that of Micro TTV isolate microTTV-HD14.2 in sample P7 (Figure S5). Several contigs were also assembled *de novo* and annotated as TTVs (BLASTx against the non-redundant NCBI database, E-value<1e^−05^) (Table S6). The presence of contig 127, contig 129 and contig 64 was verified by standard PCR in sample P7, which, according to *in silico* analyses, had the greatest abundance of TTVs. PCRs targeting contig 127 and contig 64 showed non-specific amplification, while PCR targeting contig 129 yielded only a specific PCR product that was further verified by Sanger sequencing. This contig contained one ORF that matched the ORF1 of torque teno virus and one GC-rich region (85.7%), typical of TTV genome organization [19], [28], [29] (Figure 4). Torque teno virus presence in the same sample was further verified by a nested PCR targeting the TTV UTR region, which is conserved among all TTV genotypes and genetic groups. The PCR result was positive and specific.

### Bacteriophages

GAAS analysis showed the presence of bacteriophages in 3 metagenomes (P1, P3, P4) with relative abundances of 4.74%, 33.69% and 49.32%, respectively. The total number of bacteriophage-related reads recovered for P1, P3 and P4 was 34, 91 and 455 sequences, respectively (BLASTX search against the Viral Genome database, E-value<1e^−05^). *Enterobacteria* phages, *Pseudomonas* phages, *Staphylococcus* phages, *Streptococcus* phages, *Pseudomonas* phages and *Burkholderia* phages were among the most represented bacteriophage-related sequences (Table 3). Moreover 30 contigs matching the *Escherichia* phage ECML-117 were assembled from sample P4. Also, sequences related to the bacterial hosts of detected bacteriophages were found, including a few sequences matching 16S rDNA (Table 3). Specific PCRs were performed on P1, P3 and P4 samples to verify the presence of the bacteriophage sequences recovered *in silico*. We used primers targeting the genomes of the bacteriophages of interest rather than primers designed specifically from the recovered metagenomic sequences. We performed a PCR targeting *Enterobacteria* phage lambda in sample P1 and targeting *Staphylococcus* phages of groups 3A-like and 11A-like and *Streptococcus* prophage EJ-1 in sample P3. For sample P4, we performed PCRs targeting Stx converting phage II, *Enterobacteria* phage BP-4795, *Pseudomonas* phage F8, *Pseudomonas* phage LMA2, *Pseudomonas* phage LBL3, *Burkholderia amphibaria* phage BcepF1 and *Enterobacteria* phage P1. Non-specific amplification was observed for *Enterobacteria* phage lambda in sample P1, for *Streptococcus* prophage EJ-1 in sample P3 and for Stx converting phage II, *Enterobacteria* phage BP-4795, *Pseudomonas* phage F8, *Pseudomonas* phage LMA2, *Pseudomonas* phage LBL3, *Burkholderia amphibaria* phage BcepF1 and *Enterobacteria* phage P1 in sample P4 (Table S7). For sample P3, we obtained unique amplification products at the expected length (Table S7) for *Staphylococcus* phages of groups 3A-like and 11A-like. The PCR products were sequenced and their correct identification was confirmed by Sanger technology.

**Table 3:** Detection of bacteriophage sequences in metagenomes P1, P3 and P4 and of their corresponding bacterial hosts.

Sample	Bacteriophage^a^	Number of bacteriophage reads^b^	Number of bacteriophage contigs^c^	Bacterial host^d^	Reads matching bacterial genomes^e^	Number of 16S rDNA matching reads^f^
P1	*Enterobacteria* phages	19	0	*Enterobacteria*	Yes	0
P3	*Streptococcus* phages	18	0	*Streptococci*	Yes	0
	*Staphylococcus* phages	12	0	*Staphylococci*	Yes	1
P4	*Pseudomonas* phages	170	0	*Pseudomonas*	Yes	3
	*Burkholderia* phages	76	0	*Burkholderia*	Yes	0
	*Enterobacteria* phages	49	30	*Enterobacteria*	yes	9

a Only the most abundant bacteriophage types (according to GAAS analysis) are reported. Bacteriophages are grouped according to the genus of their putative bacterial host.

b Number of reads matching the corresponding bacteriophage according to BLASTX against the RefSeq Viral Genomes database, E-value<1e^−05^.

c Number of contigs matching the corresponding bacteriophage according to BLASTX against the non-redundant NCBI database, E-value<1e^−05^.

d Only the bacterial hosts corresponding to the most abundant bacteriophage types (according to GAAS analysis) are reported.

e Presence (“yes”) or absence (“no”) of sequences matching the genome of the corresponding bacterial hosts (MG-RAST annotation based on BLAT search on the SEED database, E-value<1e-05).

f Number of sequences matching the corresponding bacterial 16S rRNA (BLASTN against the Greengenes database, E-value<1e-05, minimum alignment length 50 bp).

## Discussion

In this study, we successfully generated viral metagenomes from human pericardial fluids. In the positive control patient, we reconstructed almost the entire genome of HHV3 (human herpesvirus 3), which had been previously detected by molecular diagnostic analyses. HHV3 infections are usually self-limiting, although pericarditis has been previously described as a rare complication [30], [31]. Serendipitously, the virome generated for the positive control also revealed the presence of 4 different HPV genotypes. To our knowledge, HPV has never been associated with pericarditis. HPVs are known to be broadly distributed in human body (respiratory tract, gut, skin, blood and genitor-urinary tract) and although HPV particles might be introduced from skin during sample collection, the large number of reads related to these viruses does not support this hypothesis. The presence of multiple concomitant viral species/genotypes may be explained by the fact that this patient suffered a systemic lupus erythematosus and immunosuppressive medication increased permissiveness to viral infections.

Two patients and the negative controls viromes displayed a high relative abundance of *Retroviridae*, which may result from the residual presence of host DNA, although VLPs were DNAse and RNAse digested to eliminate peripheral contaminations. The presence of contaminating sequences is a well-known issue in the generation of host-associated viral metagenomes. In previous viral metagenomic studies, the proportion of human-related sequences represented 24%–36% of the virome [7]. These sequences represented up to 34% of generated sequences even when stringent protocols, such as filtration followed by cesium chloride density gradient purification, were adopted for the purification of viral particles [32]. In our work, abundant contamination from residual host DNA was detected in 3 patients, which may reflect the paucity of amplifiable VLPs in these samples.

Five patients metagenomes and negative control N2 had sequences that matched *Anelloviridae*, particularly torque teno viruses. Torque teno viruses were originally identified in the serum of a patient with idiopathic hepatitis [33]. Since then, they have been demonstrated to be highly ubiquitous in the human population [34]–[36] and have been found in sera, blood and multiple tissues, as well as in several organs and cells, including liver and peripheral blood mononuclear cells [19], [37], [38]. Our virome analysis showed that up to several different genotypes of TTV could be retrieved concomitantly in an individual sample. Co-infection by different TTV species or isolates is common and has been hypothesized as necessary for productive infection [38]–[41]. In sample P7, the number of TTV sequences was high and comparable to that of HHV sequences in the positive control. In addition, the viral community composition of P7 was dominated by torque teno viruses and other *Anelloviridae*. These two observations suggest the occurrence of viremia by torque teno virus in P7. To our knowledge, this is the first time that the presence of TTVs has been described in pericardial fluids. However, no evidence of the TTV association with idiopathic pericarditis can be provided, nor has an association with this disease been described in literature.

Finally, 3 patients showed the presence of sequences related to bacteriophages. Although we cannot exclude that they could represent a contamination from the skin this is highly unlikely, considering the strictly sterile conditions under which samples were collected. Moreover, bacteriophages are known to be present in the human body, as it was first discovered by Felix d'Herelle almost 90 years ago [42]. Metagenomic studies have revealed the prevalence of bacteriophages in viral communities associated with different human samples such as saliva [43], sputum [32], feces [44] and oropharyngeal samples [45]. In this study, we describe the occurrence of bacteriophages in pericardial fluids. The presence of bacteriophages in a closed system such as the pericardium indicates that phages may circulate in the human body. This hypothesis is supported a previous study showing that phages circulate in the blood of patients with septicemia [46]. We detected bacteriophages infecting *Enterobacteria, Pseudomonas, Staphylococci, Streptococci* and *Burkholderia*. However, except for sample P4, the corresponding bacteria were not detected in the samples during previous hospital routine screenings. Sample P4 came from a sudden infant death protocol and culture of the pericardial fluid showed a polymicrobial infection (*Escherichia* and *Pseudomonas* species) as a probable consequence of post-mortem bacterial invasion. In accordance with this result, the viral metagenomic analysis showed that the viral community was dominated by bacteriophage sequences related to these bacterial hosts. We were able to reconstruct 30 contigs that matched an *Escherichia coli* phage. Viral metagenomes from patients P1 and P3 also contained bacteriophage sequences, but the corresponding bacteria were not detected in the samples by culture. Early antibiotic therapy has been proposed as one factor responsible for the negative results of blood- and joint-cultures in the case of *S. aureus* endocarditis and osteoarticular infections, respectively [47], [48]. The presence of bacteriophages in human samples may represent traces of an ancient bacterial infection or indicate a current one in which the amount of bacterial DNA is below the detection threshold of the diagnostic method used due to antibiotic treatment and/or clearance by the host immune system. As bacteriophages are estimated to outnumber their bacterial hosts by a ratio of approximately 10 to 1 depending to the environmental niche [49], [50], the detection of bacteriophages could thus be more sensitive than detection of their bacterial hosts. However in this study, an undetected bacterial infection cannot be considered the etiology of pericarditis cases, as bacterial pericarditis presents specific symptoms (*e.g.* purulence), which were not showed by the patients.

In conclusion, the data provided in this study allow assessment of the DNA virome in human pericardial fluids. In particular they provide for the first time evidence for the presence of torque teno viruses and papillomaviruses in pericardial fluids.

## Supporting Information

Figure S1 **Reconstruction of the human herpesvirus 3 genome from the positive control virome.** The HHV3 reference genome was reconstructed by mapping the metagenomic reads generated from the positive control sample. Open Reading Frames of the reference genome (yellow arrows), reference coverage (black line) and coverage depth (pink shadow) are shown along the genome.
(PDF)

Figure S2 **Reconstruction of the human papillomavirus genomes detected in the positive control virome.** The reference genomes of human papillomavirus type 12, type 50 and type 80 as well as that of human papillomavirus isolate 915 F 06 002 KN1 were reconstructed by mapping the metagenomic reads generated from the positive control sample. Open Reading Frames of the reference genome (yellow arrows), reference coverage (black line) and coverage depth (pink shadow) are shown.
(PDF)

Figure S3 **Reconstruction of the torque teno virus genome detected in patient P2 virome.** The reference genome of torque teno virus clone Saf-09 was reconstructed by mapping from sample P2. The open reading frames of the reference genome (yellow arrows), reference coverage (black line) and coverage depth (pink shadow) are shown.
(PDF)

Figure S4 **Reconstruction of the torque teno virus genomes detected in patient P6 virome.** The reference genome of torque teno virus strain SIA 09 and torque teno virus 24 Saa-01 were reconstructed by mapping from sample P6. The open reading frames of the reference genome (yellow arrows), reference coverage (black line) and coverage depth (pink shadow) are shown.
(PDF)

Figure S5 **Reconstruction of the torque teno virus genomes detected in patient P7 virome.** The reference genome of torque teno virus isolate TTVyon-LC011, torque teno virus 29, micro torque teno virus, isolate microTTV-HD14.2, torque teno virus TTV-HD14 h and torque teno virus isolate JT41F were reconstructed by mapping from sample P7. The open reading frames of the reference genome (yellow arrows), reference coverage (black line) and coverage depth (pink shadow) are shown.
(PDF)

Table S1 **Primers used in this study.** Different PCR primers were used to confirm the presence of a selected viral species detected *in silico*. For each primer pair, the organism and sequence targeted, primer names, expected amplicon size, sequences and method used to design the primers are listed.
(DOC)

Table S2 **High-throughput sequencing output.** For each sample, the total number of reads generated, the number of reads remaining after duplicate elimination (preprocessing) and the average length of the preprocessed reads are reported.
(DOC)

Table S3 **Metagenomes assembly.** For each sample, the total number of contigs (“Contigs”), the number of contigs spanning more than 1,500 bp (“Large contigs”) and the percentage of reads assembled into contigs (“Assembled reads”) are reported.
(DOC)

Table S4 **Reconstruction of human papillomavirus genomes by mapping of the positive control virome.** For each reference genome, we reported the length of the consensus sequence reconstructed by mapping, the number of reads mapped, the average depth coverage and the proportion of the reference genome that was reconstructed.
(DOC)

Table S5 ***Anelloviridae*** **detected in the viromes.** For each virome, the absolute number of reads matching each identified species of *Anelloviridae* (BLASTX search against the non-redundant NCBI database, E-value<1e^-05^) is listed. Species are grouped according to the genus to which they belong.
(DOC)

Table S6 **Reconstructed contigs matching** ***Anelloviridae*** **.** For each contig, we list the sample from which it was assembled, the length, the number of reads assembled in the contig and the GC content. The best BLAST hit (BLASTX against the non-redundant NCBI database, E-value<1e^-05^) is also shown, as well as the hit alignment parameters: E-value, percentage of identity, and alignment length.
(DOC)

Table S7 **Standard PCRs performed to confirm the presence of the bacteriophages detected** ***in silico*** **.** The sample, primers used, bacteriophage targeted and results of the amplification are listed.
(DOC)
