# Supplementary figures and images for: Viral Communities Associated with Human Pericardial Fluids in Idiopathic Pericarditis
Source: PLoS One. 2014 Apr 1;9(4):e93367. doi: 10.1371/journal.pone.0093367 (PMC3972187; doi:10.1371/journal.pone.0093367)

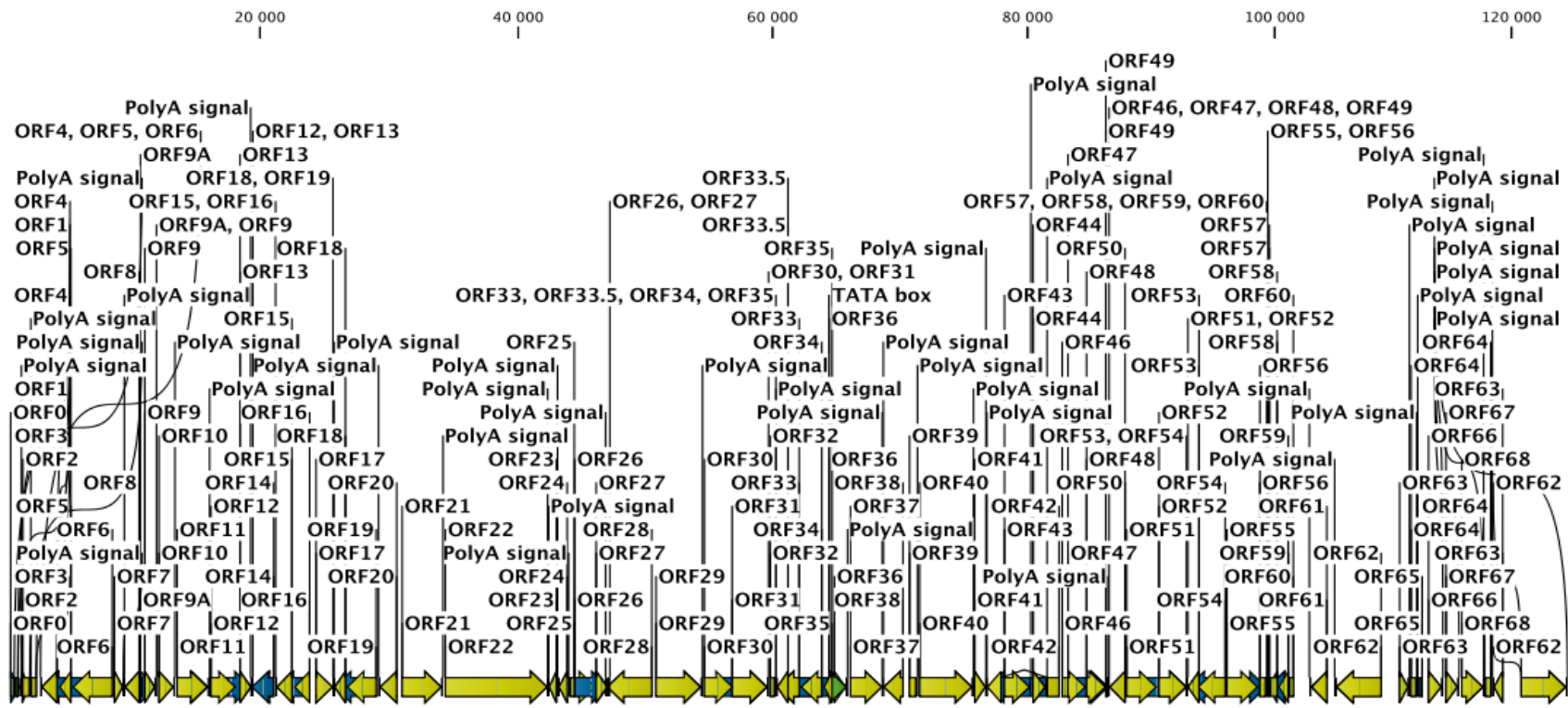

Human Herpesvirus 3

Reference coverage: 98% (122502 bp)

Average coverage depth: 18.93

Supplement: Figure S1 — Reconstruction of the human herpesvirus 3 genome from the positive control virome. The HHV3 reference genome was reconstructed by mapping the metagenomic reads generated from the positive control sample. Open Reading Frames of the reference genome (yellow arrows), reference coverage (black line) and coverage depth (pink shadow) are shown along the genome. (PDF) [file pone.0093367.s001.pdf]

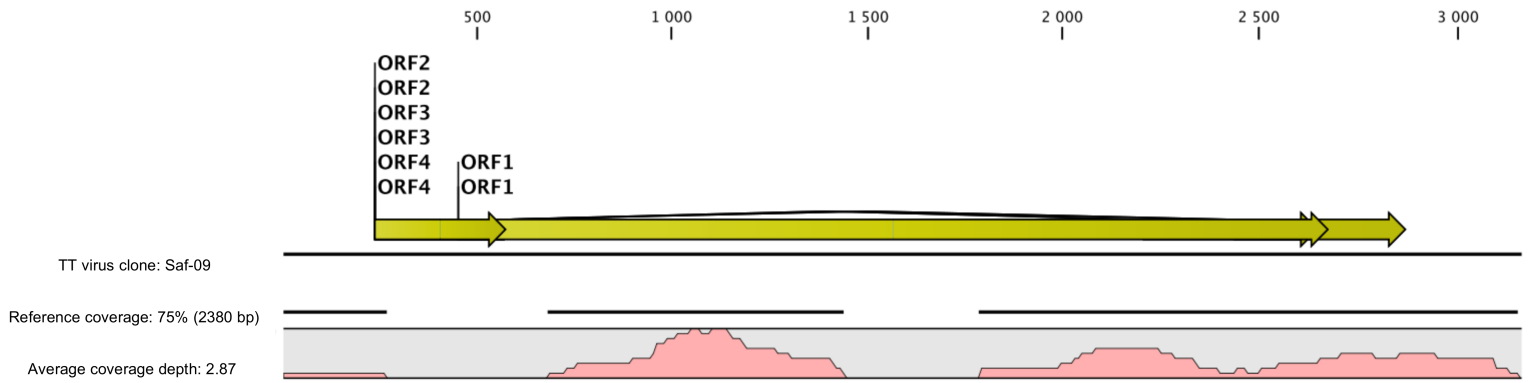

Supplement: Figure S3 — Reconstruction of the torque teno virus genome detected in patient P2 virome. The reference genome of torque teno virus clone Saf-09 was reconstructed by mapping from sample P2. The open reading frames of the reference genome (yellow arrows), reference coverage (black line) and coverage depth (pink shadow) are shown. (PDF) [file pone.0093367.s003.pdf]

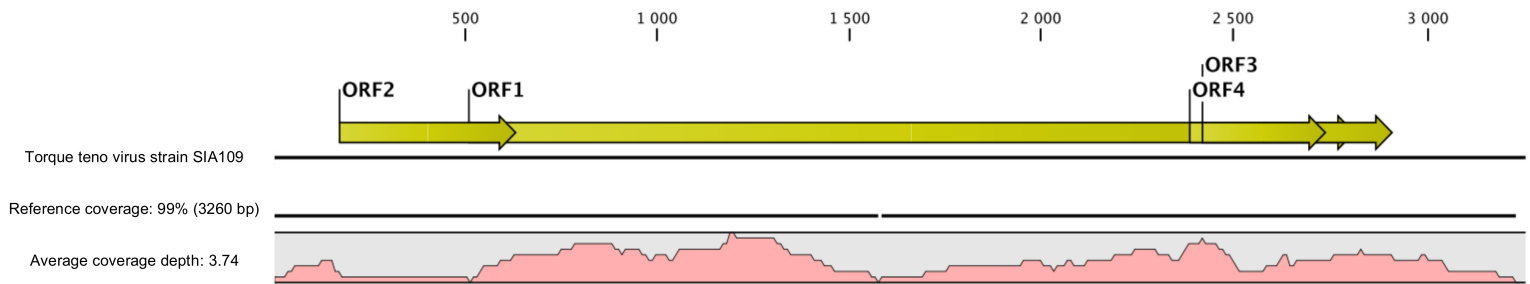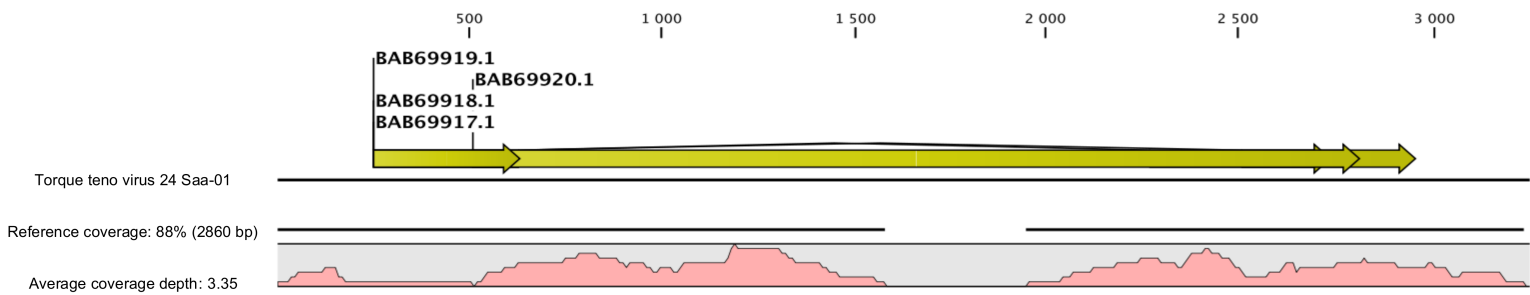

Supplement: Figure S4 — Reconstruction of the torque teno virus genomes detected in patient P6 virome. The reference genome of torque teno virus strain SIA 09 and torque teno virus 24 Saa-01 were reconstructed by mapping from sample P6. The open reading frames of the reference genome (yellow arrows), reference coverage (black line) and coverage depth (pink shadow) are shown. (PDF) [file pone.0093367.s004.pdf]

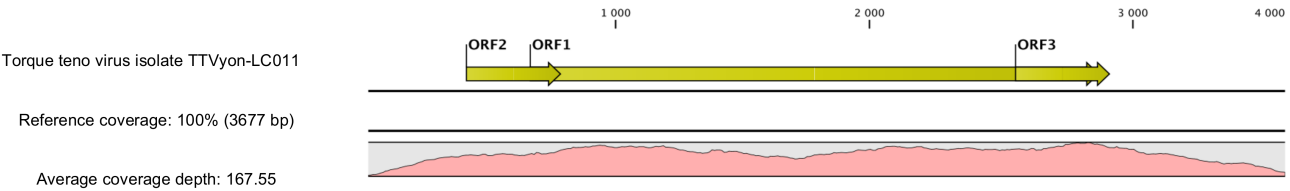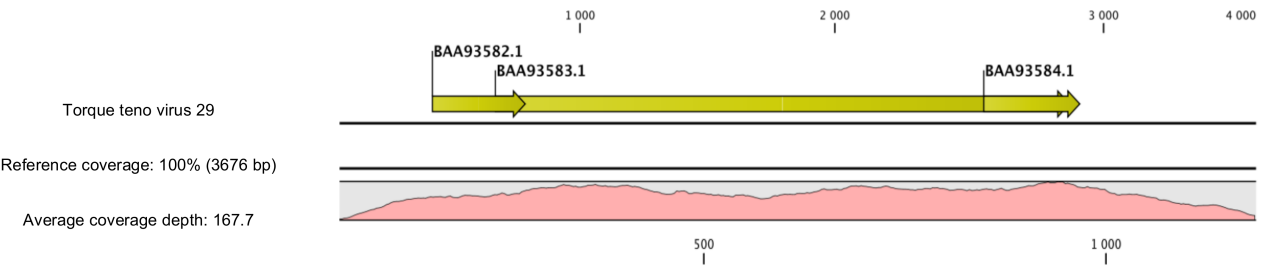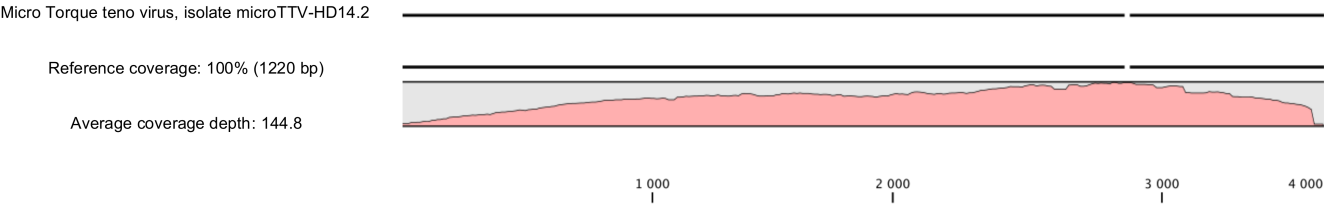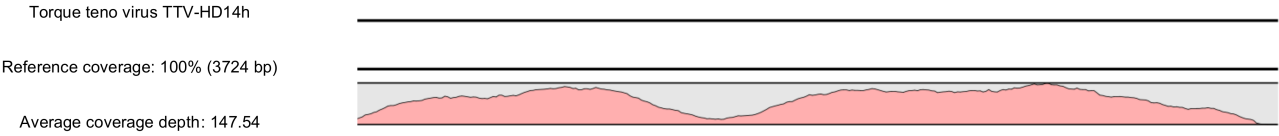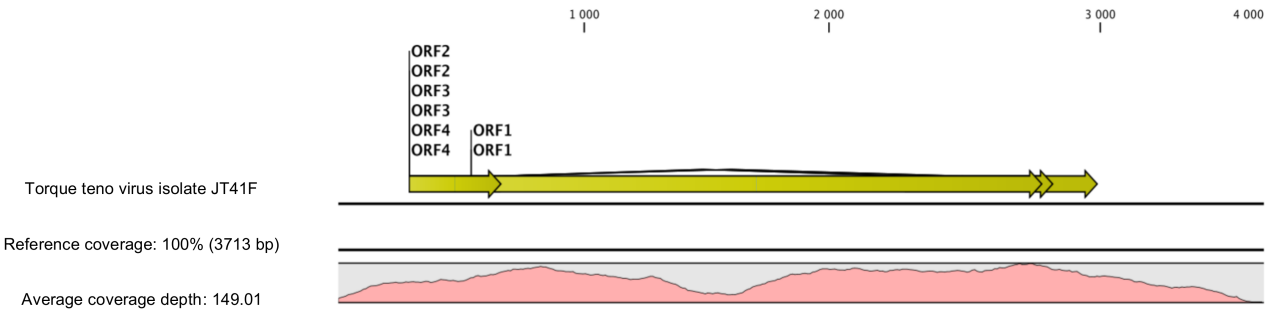

Supplement: Figure S5 — Reconstruction of the torque teno virus genomes detected in patient P7 virome. The reference genome of torque teno virus isolate TTVyon-LC011, torque teno virus 29, micro torque teno virus, isolate microTTV-HD14.2, torque teno virus TTV-HD14 h and torque teno virus isolate JT41F were reconstructed by mapping from sample P7. The open reading frames of the reference genome (yellow arrows), reference coverage (black line) and coverage depth (pink shadow) are shown. (PDF) [file pone.0093367.s005.pdf]
