# Supplementary material for: Viral Communities Associated with Human Pericardial Fluids in Idiopathic Pericarditis
Source: PLoS One. 2014 Apr 1;9(4):e93367. doi: 10.1371/journal.pone.0093367 (PMC3972187; doi:10.1371/journal.pone.0093367)
Supplement: Figure S2 — Reconstruction of the human papillomavirus genomes detected in the positive control virome. The reference genomes of human papillomavirus type 12, type 50 and type 80 as well as that of human papillomavirus isolate 915 F 06 002 KN1 were reconstructed by mapping the metagenomic reads generated from the positive control sample. Open Reading Frames of the reference genome (yellow arrows), reference coverage (black line) and coverage depth (pink shadow) are shown. (PDF) [file pone.0093367.s002.pdf]

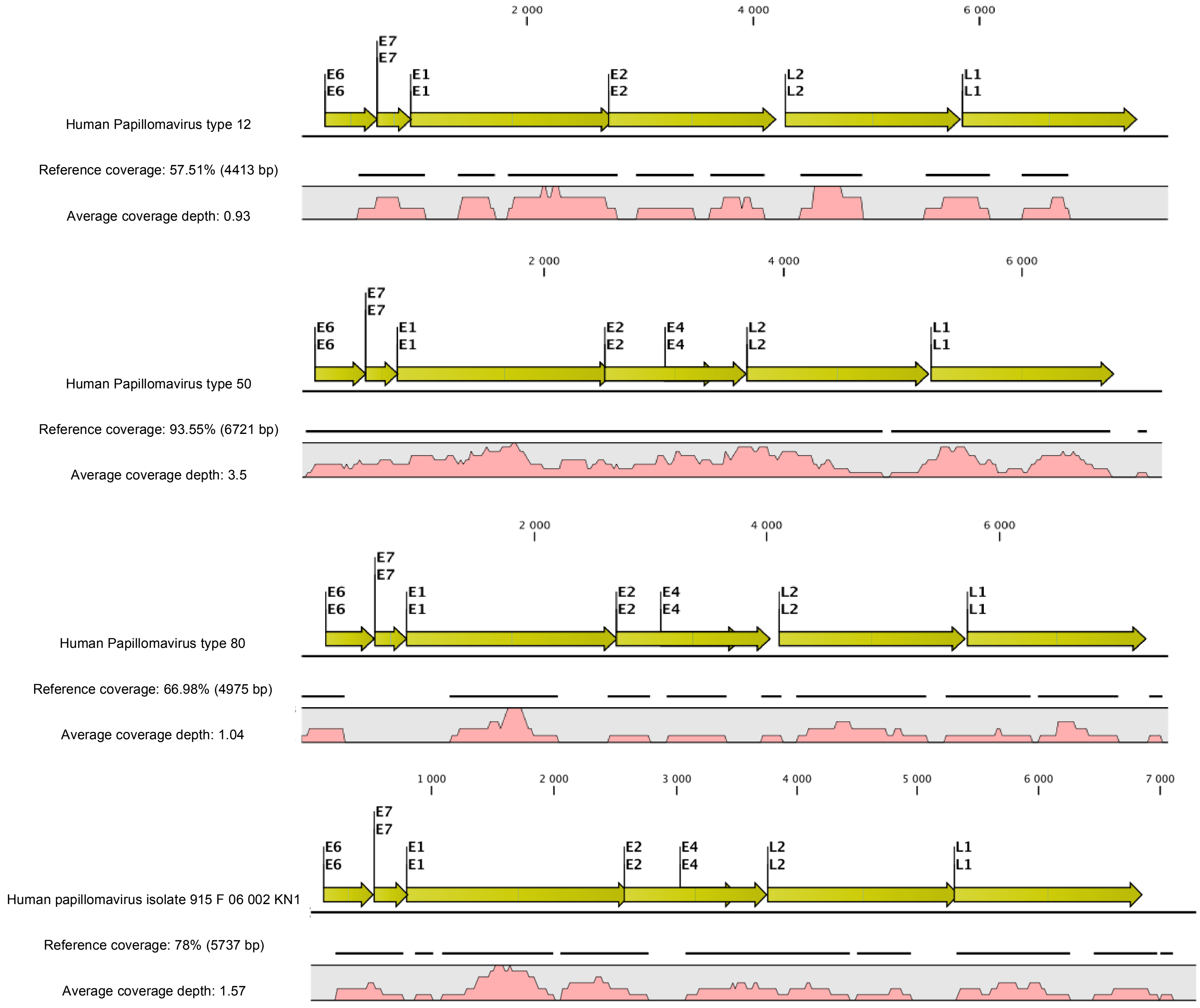

2 000
4 000
6 000
E6
E7
E1
E2
L2
L1
Human Papillomavirus type 12
Reference coverage: 57.51% (4413 bp)
Average coverage depth: 0.93
2 000
4 000
6 000
E6
E7
E1
E2
E4
L2
L1
Human Papillomavirus type 50
Reference coverage: 93.55% (6721 bp)
Average coverage depth: 3.5
2 000
4 000
6 000
E6
E7
E1
E2
E4
L2
L1
Human Papillomavirus type 80
Reference coverage: 66.98% (4975 bp)
Average coverage depth: 1.04
1 000
2 000
3 000
4 000
5 000
6 000
7 000
E6
E7
E1
E2
E4
L2
L1
Human papillomavirus isolate 915 F 06 002 KN1
Reference coverage: 78% (5737 bp)
Average coverage depth: 1.57
