# Supplementary material for: Viral Communities Associated with Human Pericardial Fluids in Idiopathic Pericarditis
Source: PLoS One. 2014 Apr 1;9(4):e93367. doi: 10.1371/journal.pone.0093367 (PMC3972187; doi:10.1371/journal.pone.0093367)
Supplement: Table S1 — Primers used in this study. Different PCR primers were used to confirm the presence of a selected viral species detected in silico. For each primer pair, the organism and sequence targeted, primer names, expected amplicon size, sequences and method used to design the primers are listed. (DOC) [file pone.0093367.s006.doc]

**Table S1. Primers used in this study.** Different PCR primers were used to confirm the presence of a selected viral species detected *in silico*. For each primer pair, the organism and sequence targeted, primer names, expected amplicon size, sequences and method used to design the primers are listed.

| **Organism** | **Targeted sequence** | **Primer name** | **Expected product size (bp)** | **Primer sense (5' → 3')** | **Primer antisense (5' → 3')** | **Primer design** |
| --- | --- | --- | --- | --- | --- | --- |
| TTV | UTR-region | Internal primers NG472, NG352; | 91 | GCGTCCCGWGGGCGGGTGCCG [W = A or T] | GAGCCTTGCCCATRGCCCGGCCAG [R = A or G] | Literature [18] |
|  |  | External primers NG473, NG351 | 71 | CGGGTGCCGDAGGTGAGTTTACAC [D = G, A or T] | CCCATRGCCCGGCCAGTCCCGAGC | Literature [18] |
| TTV | TTV-matching contig 127 (sample P7) | 127L, 127R | 819 | CCCATGGAGCAGATGTCTTT | CAGGGCTAGACCAGCTCAAC | Primer3 [19] |
| TTV | TTV-matching contig 129 (sample P7) | 129L, 129R | 820 | CCTGCTGCTCTGGTATCCAT | TTACACACGACTGGGGCATA | Primer3 [19] |
| TTV | TTV-matching contig 164 (sample P7) | 164L, 164R | 783 | AGCGGAAGGAAGTCACAAGA | GGACCCCCTCCTGTCATAAT | Primer3 [19] |
| *Staphylococcus* phage 47, 42E, phi 12, tp310-2 | tail fiber | SGA1, SGA2 | 744 | TATCAGGCGAGAATTAAGGG | CTTTGACATGACATCCGCTTGAC | Literature [20] |
| *Staphylococcus* phage 96, phi ETA3 | hypothetical tail protein | SGB1, SGB2 | 405 | ACTTATCCAGGTGGYGTTATTG | TGTATTTAATTTCGCCGTTAGTG | Literature [20] |
| *Enterobacteria* phage P1 | tyrosine recombinases | EphP1, EphP2 | 447 | TGCTTATAACACCCTGTTACGTAT | CAGCCACCAGCTTGCATGATC | Literature [21] |
| *Enterobacteria* phage lambda | minor capsid protein | EphL1, EphL2 | 378 | TCATCGCCCGTGTGCGTGAC | GCCTGCGAAGCAGTGGCTGA | Primer-BLAST (www.ncbi.nlm.nih.gov/tools/primer-blast/) |
| Stx2 converting phage II, *Enterobacteria* phage BP-4795 | NinG protein | RF41, RF42 | 453 | CAAATCAGTGGTGGTGCT | TGGCGGTTATGGTTCG | PhiSigns [22] |
| *Pseudomonas* phage F8,LMA2, LBL3 | phage protein | Pph1, Pph2 | 458 | GTTGCTGCGGATTTGA | GTCTGCGGCGTTCTGG | PhiSigns [22] |
| *Burkholderia ambifaria* phage BcepF1*, Streptococcus* prophage EJ-1 | hypothetical protein BcepF1.101 | Bph1, Bph2 | 173 | CGTAAATGCCTTGACCGTTT | CATCCAGTGGGTGAACACAG | Primer3 [19] |
| *Streptococcus* prophage EJ-1 | minor head protein | StrePh1, StrePh2 | 954 | CTAAAACGCATTACGGGGCG | GCGGACTCTGTCCTTTTCCA | Primer-BLAST (www.ncbi.nlm.nih.gov/tools/primer-blast/) |
