# Supplementary material for: Viral Communities Associated with Human Pericardial Fluids in Idiopathic Pericarditis
Source: PLoS One. 2014 Apr 1;9(4):e93367. doi: 10.1371/journal.pone.0093367 (PMC3972187; doi:10.1371/journal.pone.0093367)
Supplement: Table S2 — High-throughput sequencing output. For each sample, the total number of reads generated, the number of reads remaining after duplicate elimination (preprocessing) and the average length of the preprocessed reads are reported. (DOC) [file pone.0093367.s007.doc]

**Table S2. High-throughput sequencing output.** For each sample, the total number of reads generated, the number of reads remaining after duplicate elimination (preprocessing) and the average length of the preprocessed reads are reported.

| **Sample** | **Total number of reads** | **Number of reads after preprocessing** | **Average read length after preprocessing (bp)** |
| --- | --- | --- | --- |
| P1 | 41711 | 26631 | 334.82 |
| P2 | 34452 | 30595 | 311.79 |
| P3 | 33909 | 16297 | 359.62 |
| P4 | 35817 | 23843 | 341.57 |
| P5 | 29614 | 27884 | 287.77 |
| P6 | 26369 | 23761 | 246.22 |
| P7 | 26220 | 23788 | 260.05 |
| P8 | 28691 | 23360 | 280.66 |
| Positive control | 29812 | 16419 | 361.8 |
| Pool negative controls N1 | 26562 | 23060 | 336.84 |
| Negative control N2 | 23304 | 22033 | 324 |
