# Supplementary material for: Viral Communities Associated with Human Pericardial Fluids in Idiopathic Pericarditis
Source: PLoS One. 2014 Apr 1;9(4):e93367. doi: 10.1371/journal.pone.0093367 (PMC3972187; doi:10.1371/journal.pone.0093367)
Supplement: Table S3 — Metagenomes assembly. For each sample, the total number of contigs (“Contigs”), the number of contigs spanning more than 1,500 bp (“Large contigs”) and the percentage of reads assembled into contigs (“Assembled reads”) are reported. (DOC) [file pone.0093367.s008.doc]

**Table S3. Metagenomes assembly.** For each sample, the total number of contigs (“Contigs”), the number of contigs spanning more than 1,500 bp (“Large contigs”) and the percentage of reads assembled into contigs (“Assembled reads”) are reported.

| **Sample** | **Contigs** | **Large contigs** | **Assembled reads (%)** |
| --- | --- | --- | --- |
| P1 | 260 | 42 | 60.94 |
| P2 | 415 | 95 | 43.9 |
| P3 | 83 | 13 | 76.66 |
| P4 | 282 | 19 | 44.71 |
| P5 | 32 | 2 | 2.62 |
| P6 | 226 | 8 | 16.93 |
| P7 | 97 | 3 | 12.43 |
| P8 | 233 | 17 | 35.11 |
| Positive control | 79 | 16 | 67.66 |
| Pool negative controls N1 | 157 | 18 | 22.42 |
| Negative control N2 | 136 | 7 | 10.61 |
