# Supplementary material for: Viral Communities Associated with Human Pericardial Fluids in Idiopathic Pericarditis
Source: PLoS One. 2014 Apr 1;9(4):e93367. doi: 10.1371/journal.pone.0093367 (PMC3972187; doi:10.1371/journal.pone.0093367)
Supplement: Table S4 — Reconstruction of human papillomavirus genomes by mapping of the positive control virome. For each reference genome, we reported the length of the consensus sequence reconstructed by mapping, the number of reads mapped, the average depth coverage and the proportion of the reference genome that was reconstructed. (DOC) [file pone.0093367.s009.doc]

**Table S4. Reconstruction of human papillomavirus genomes by mapping of the positive control virome.** For each reference genome, we reported the length of the consensus sequence reconstructed by mapping, the number of reads mapped, the average depth coverage and the proportion of the reference genome that was reconstructed.

| **Reference genome** | **Consensus length (bp)** | **Number of reads mapped** | **Average depth coverage** | **% of reference covered** |
| --- | --- | --- | --- | --- |
| Human papillomavirus type 12 | 4413 | 20 | 0.93 | 57.51 |
| Human papillomavirus type 50 | 6721 | 69 | 3.5 | 93.55 |
| Human papillomavirus type 80 | 4975 | 23 | 1.04 | 66.98 |
| Human papillomavirus isolate 915 F 06 002 KN1 | 5737 | 32 | 1.57 | 78 |
