# Supplementary material for: Viral Communities Associated with Human Pericardial Fluids in Idiopathic Pericarditis
Source: PLoS One. 2014 Apr 1;9(4):e93367. doi: 10.1371/journal.pone.0093367 (PMC3972187; doi:10.1371/journal.pone.0093367)
Supplement: Table S5 — Anelloviridae detected in the viromes. For each virome, the absolute number of reads matching each identified species of Anelloviridae (BLASTX search against the non-redundant NCBI database, E-value<1e-05) is listed. Species are grouped according to the genus to which they belong. (DOC) [file pone.0093367.s010.doc]

**Table S5. *Anelloviridae* detected in the viromes.** For each virome, the absolute number of reads matching each identified species of *Anelloviridae* (BLASTX search against the non-redundant NCBI database, E-value<1e-05) is listed. Species are grouped according to the genus to which they belong.

| **Genus** | **Species** | **P1** | **P2** | **P3** | **P4** | **P5** | **P6** | **P7** | **P8** | **Positive control** | **Pool negative controls N1** | **Negative control N2** |
| --- | --- | --- | --- | --- | --- | --- | --- | --- | --- | --- | --- | --- |
| Alfatorquevirus | Torque teno virus 1 | 0 | **4** | 0 | 0 | 0 | 0 | 0 | 0 | 0 | 0 | 0 |
|  | Torque teno virus 10 | 0 | 0 | 0 | 0 | 0 | 0 | **43** | 0 | 0 | 0 | 0 |
|  | Torque teno virus 16 | 0 | **7** | 0 | 0 | 0 | 0 | 0 | 0 | 0 | 0 | 0 |
|  | Torque teno virus 19 | 0 | **1** | 0 | 0 | 0 | 0 | 0 | 0 | 0 | 0 | 0 |
|  | Torque teno virus 21 | 0 | **13** | 0 | 0 | 0 | 0 | 0 | 0 | 0 | 0 | 0 |
|  | Torque teno virus 23 | 0 | **3** | 0 | 0 | 0 | **1** | 0 | 0 | 0 | 0 | 0 |
|  | Torque teno virus 24 | 0 | **1** | 0 | 0 | 0 | **12** | 0 | 0 | 0 | 0 | 0 |
|  | Torque teno virus 27 | 0 | 0 | 0 | 0 | 0 | 0 | **2** | 0 | 0 | 0 | 0 |
|  | Torque teno virus 28 | 0 | 0 | 0 | 0 | 0 | 0 | **1** | 0 | 0 | 0 | 0 |
|  | Torque teno virus 29 | 0 | 0 | 0 | 0 | 0 | 0 | **366** | 0 | 0 | 0 | 0 |
| Betatorquevirus | Torque teno mini virus 1 | 0 | 0 | 0 | 0 | 0 | 0 | **1** | 0 | 0 | 0 | 0 |
|  | Torque teno mini virus 2 | 0 | **2** | 0 | 0 | 0 | 0 | 0 | 0 | 0 | 0 | 0 |
|  | Torque teno mini virus 3 | 0 | 0 | 0 | 0 | 0 | 0 | **1** | 0 | 0 | 0 | 0 |
| Gammatorquevirus | Torque teno midi virus 2 | **1** | 0 | 0 | 0 | 0 | 0 | 0 | 0 | 0 | 0 | 0 |
| unclassified Anelloviridae | SEN virus | 0 | **2** | 0 | 0 | 0 | **2** | 0 | 0 | 0 | 0 | **1** |
|  | Small anellovirus | **1** | 0 | 0 | 0 | 0 | 0 | 0 | 0 | 0 | 0 | **3** |
|  | Torque teno midi virus | **18** | **4** | 0 | 0 | 0 | 0 | 0 | 0 | 0 | 0 | 0 |
|  | Torque teno virus | 0 | **71** | 0 | 0 | 0 | **41** | **1214** | 0 | 0 | 0 | **13** |
|  | TTV-like mini virus | 0 | 0 | **3** | 0 | 0 | 0 | 0 | 0 | 0 | 0 | 0 |
