# Supplementary material for: Viral Communities Associated with Human Pericardial Fluids in Idiopathic Pericarditis
Source: PLoS One. 2014 Apr 1;9(4):e93367. doi: 10.1371/journal.pone.0093367 (PMC3972187; doi:10.1371/journal.pone.0093367)
Supplement: Table S6 — Reconstructed contigs matching Anelloviridae . For each contig, we list the sample from which it was assembled, the length, the number of reads assembled in the contig and the GC content. The best BLAST hit (BLASTX against the non-redundant NCBI database, E-value<1e-05) is also shown, as well as the hit alignment parameters: E-value, percentage of identity, and alignment length. (DOC) [file pone.0093367.s011.doc]

**Table S6. Reconstructed contigs matching *Anelloviridae*.** For each contig, we list the sample from which it was assembled, the length, the number of reads assembled in the contig and the GC content. The best BLAST hit (BLASTX against the non-redundant NCBI database, E-value<1e-05) is also shown, as well as the hit alignment parameters: E-value, percentage of identity, and alignment length.

| **Sample** | **Contig ID** | **Contig length (bp)** | **Number of reads** | **GC content (%)** | **Number of ORFs** | **Best BLAST hit** | **E-value** | **Percentage identity** | **Alignment length (aa)** |
| --- | --- | --- | --- | --- | --- | --- | --- | --- | --- |
| P1 | contig00011 | 482 | 5 | 36.1 | 1 | hypothetical protein ORF1-like [Torque teno midi virus] | 3e-33 | 62.40 | 125 |
|  | contig00088 | 455 | 7 | 38.9 | 1 | hypothetical protein ORF1-like [Torque teno midi virus] | 3e-24 | 39.62 | 159 |
|  | contig00175 | 517 | 3 | 47 | 1 | hypothetical protein ORF2-like [Torque teno midi virus] | 5e-23 | 69.86 | 73 |
| P2 | contig00198 | 3532 | 96 | 48.9 | 2 | ORF1 [Torque teno virus] | 0 | 69.46 | 681 |
| P6 | contig00002 | 860 | 13 | 57.1 | 1 | unnamed protein product ORF2-like [Torque teno virus 24] | 4e-19 | 60.00 | 80 |
|  | contig00008 | 1011 | 17 | 49.3 | 3 | ORF1 [Torque teno virus] | 2e-79 | 63.25 | 117 |
|  | contig00188 | 1060 | 18 | 45.9 | 2 | ORF1 [Torque teno virus] | 2e-171 | 91.03 | 234 |
| P7 | contig00001 | 526 | 4 | 51 | 1 | hypothetical protein TTV10_gp4 ORF1-like [Torque teno virus 10] | 6e-52 | 98.67 | 75 |
|  | contig00064 | 2668 | 1358 | 56.5 | 4 | ORF1 [Torque teno virus] | 6e-142 | 87.40 | 262 |
|  | contig00127 | 3572 | 276 | 52.2 | 3 | ORF1 [Torque teno virus] | 0 | 89.52 | 668 |
|  | contig00129 | 981 | 739 | 49.4 | 1 | ORF1 [Torque teno virus] | 8e-176 | 88.27 | 324 |
