# Supplementary material for: Viral Communities Associated with Human Pericardial Fluids in Idiopathic Pericarditis
Source: PLoS One. 2014 Apr 1;9(4):e93367. doi: 10.1371/journal.pone.0093367 (PMC3972187; doi:10.1371/journal.pone.0093367)
Supplement: Table S7 — Standard PCRs performed to confirm the presence of the bacteriophages detected in silico . The sample, primers used, bacteriophage targeted and results of the amplification are listed. (DOC) [file pone.0093367.s012.doc]

**Table S7. Standard PCRs performed to confirm the presence of the bacteriophages detected *in silico.*** The sample, primers used, bacteriophage targeted and results of the amplification are listed.

| **Sample** | **Primers** | **Targeted phage** | **PCR amplification** |
| --- | --- | --- | --- |
| P1 | EphL1, EphL2 | *Enterobacteria* phage Lambda | Negative |
| P3 | SGA1, SGA2 | *Staphylococcus* phages 3A-like | Positive, specific |
| P3 | SGB1, SGB2 | *Staphylococcus* phages 11A-like | Positive, specific |
| P3 | StrePh1, StrePh2 | *Streptococcus* prophage EJ-1 | Aspecific amplificates |
| P4 | RF41, RF42 | Stx converting phage II, *Enterobacteria* phage BP-4795 | Aspecific amplificates |
| P4 | Pph1, Pph2 | *Pseudomonas* phage F8, LMA2, LBL3 | Aspecific amplificates |
| P4 | Bph1, Bph2 | *Burkholderia amphibaria* phage BcepF1 | Aspecific amplificates |
| P4 | EphP1, Eph2 | *Enterobacteria* phage P1 | Aspecific amplificates |
